# *Glutamicibacter* sp. ZY1 antagonizes pathogenic *Vibrio parahaemolyticus* via iron competition

**DOI:** 10.1128/aem.00009-25

**Published:** 2025-04-24

**Authors:** Zhili Shi, Ya Li, Weibo Shi, Zhixin Mu, Qingxi Han, Weiwei Zhang

**Affiliations:** 1 School of Marine Sciences, Ningbo University 631318 , Ningbo, China; 2 Key Laboratory of Aquacultural Biotechnology Ministry of Education, Ningbo University 47862 , Ningbo, China; University of Delaware, Lewes, Delaware, USA

**Keywords:** *Glutamicibacter* sp., *Vibrio parahaemolyticus*, iron competition, antagonism

## Abstract

Probiotics are prior agents for treating bacterial infection with advantages of inhibiting pathogenic bacteria and improving immune responses of hosts, thus increasing the survival rate of cultured animals. In this study, one *Vibrio parahaemolyticus* YDE17 pathogenic to shrimp and its antagonist *Glutamicibacter* sp. ZY1 were screened, and ZY1 showed stable inhibitory effects on diverse *Vibrio* spp., especially *V. parahaemolyticus*. ZY1 secreted inhibitory substances into supernatant, and the activity of inhibitory substances did not change after being treated under different temperatures, proteinase K, and pH (6-10), which indicated that the inhibitory substances might be small molecules, which led us to trace the siderophore production. The siderophore production of YDE17 co-incubated with the cell-free supernatant of ZY1 was greater than that of YDE17 alone, which indicated that the cell-free supernatant of ZY1 created iron-limiting conditions for YDE17. This finding was confirmed by iron supplementation assays, in which the inhibitory activity of the cell-free supernatant of ZY1 on YDE17 as well as the siderophore production of YDE17 decreased in the presence of FeCl_3_. The effect of iron on inhibition was further confirmed by *in vivo* infection. The relative percent survival of ZY1 to shrimp challenged by YDE17 was 83.3%, but the survival rates of shrimp challenged with YDE17/ZY1/FeCl_3_ were similar to that of YDE17, both of which were significantly lower than the 70% survival rate of shrimps simultaneously challenged by ZY1/YDE17. Our study offers a new probiotic resource to control vibriosis, which works through iron competition with the opportunistic pathogens of *Vibrio* spp.

**IMPORTANCE:**

Bacteria belonging to *Vibrio* spp., especially *Vibrio parahaemolyticus*, are important opportunistic pathogens infecting a wide range of hosts including fish, shrimp, shellfish, and crab. Antibiotics are effective but show the disadvantages of antibiotic generation, microecology destruction, and biological toxicology; thus, new treatments of *Vibrio* infection are urgently recommended. In our present study, *Glutamicibacter* sp. ZY1, belonging to the phylum Actinomycetes, was selected and showed high inhibitory activity to inhibit *V. parahaemolyticus* pathogenic to shrimp. *Glutamicibacter* sp. ZY1 antagonized *V. parahaemolyticus* YDE17 through producing siderophore to compete for iron, based on the results of both *in vitro* and in *vivo* experiments under different iron levels. This study offers a new strategy to control *Vibrio* infection in aquaculture.

## INTRODUCTION

China is a major producer of Pacific white shrimp (*Litopenaeus vannamei*) (1), but shrimp *L. vannamei* is highly susceptible to *Vibrio* spp. infections, and mass mortality occurs during its culture (2). The pathogenic strains that could infect shrimp *L. vannamei* include *Vibrio harveyi* (3), *Vibrio anguillarum* (4), *Vibrio alginolyticus* (5), *Vibrio parahaemolyticus* (6), and *Vibrio vulnificus* (7). To prevent or cure the infection of *Vibrio* spp., antibiotics have been frequently used; however, the excess and frequent use of antibiotics has caused several hazards, including the spread of drug-resistant genes, the generation of drug-resistant bacteria, and the destruction of microbial ecosystems (8, 9). Residual antibiotics can further affect the safety of animals and humans, as well as the stability of aquatic ecosystems (9). Recent reports indicate that the use of antibiotics in aquaculture should be replaced, emphasizing the urgency for strategies with necessary improvements to ensure aquaculture and human health (10).

Probiotic is an effective tool to treat the infection caused by *Vibrio* spp. (11). The selection of probiotics that antagonize *Vibrio* spp. and the identification of the inhibitory substances of probiotics against pathogens are vital for the application of probiotics (12). Currently, probiotics that antagonize *Vibrio* spp. are *Bacillus* (13), *Pseudomonas* (14), *Lactobacillus* (15), and *Streptococcus* (16), etc. Probiotics can achieve antagonistic effects by producing active inhibitory substances; striving for nutrients, chemicals, or energy; reducing virulence through the quorum-sensing system; and improving the digestion of macro-and/or micronutrients and the immunoregulatory capacity of the host (17, 18). The active inhibitory substances include bacteriocin, siderophores, lysozyme, proteolytic enzymes, and catalases, etc. (19). Particularly, iron is a key element for the growth and survival of almost all bacteria (20). Depriving iron from pathogenic bacteria is a ubiquitous antagonistic strategy between bacteria (21). Under iron-limiting conditions, bacteria can survive by secreting siderophores, which are low-molecular-weight substances that can bind ferric iron ions and transfer iron ions into the intracellular space of bacterial cells (22). In aquaculture, probiotics that use siderophores to compete for iron with pathogens include *Streptomyces* (23), the yeast *Aureobasidium pullulans* HN 6.2 (24), *Pseudomonas aeruginosa* PA1 (25), *Bacillus* sp. (26), and *Pseudomonas* sp. (27). *Streptomyces* are bacteria belonging to the phylum Actinomycetes and are promising biocontrol agents in aquaculture. *Streptomyces* can inhibit the opportunistic pathogen belonging to *Vibrio* spp., including *V*. *vulnificus*, *V*. *alginolyticus,* and *V*. *parahaemolyticus* (28). For example, the marine *Streptomyces* S073 could inhibit pathogenic *V. parahaemolyticus*, and this inhibition was attributed to the stronger iron chelating activity of S073 through the production of carboxylate siderophores, resulting in iron-limiting conditions for the pathogen (23).

In the present study, the bacterium ZY1, which exhibited inhibitory activity against a variety of *Vibrio* spp., was screened and identified. Subsequently, the inhibitory substances produced by ZY1 were traced and characterized. The preliminary mechanism on the antagonism of ZY1 on YDE17 was explored from the viewpoint of iron competition. Our results showed that the inhibitory effect of ZY1 on YDE17 was mainly due to its siderophore production to compete for iron, but YDE17 could compensate for limited iron to a certain degree by increasing siderophore production of itself. Finally, to determine whether ZY1 could protect shrimp from infection of *V. parahaemolyticus*, artificial infections using ZY1, YDE17, and simultaneous ZY1 and YDE17 under different iron levels were performed.

## MATERIALS AND METHODS

### Bacterial isolation

The water samples were collected from a diseased Pacific white shrimp farm in Jiangmen, Guangzhou. In the laboratory, 500 mL of the water samples was filtrated using a vacuum filter (JOANLAB, China). The bacteria on the membrane were resuspended in 5 mL sterilized seawater, and 50 µL of the cell suspension was spread on a 2216E agar plate. After incubation at 28°C for 24 h, several antagonistic circles were observed. The colonies of the inhibitory bacteria and bacteria surrounding the inhibitory circles were purified and identified. The bacterial status was identified according to the sequence of 16S ribosomal RNA (16S rRNA) gene, which was amplified by PCR using the universal primers of 27F (3′-GTGCTGCAGAGAGTTTGATCCTGGCTCAG-5′) and 1492R (3′-CACGGATCCTACGGGTACCTTGTTACGACTT-5′) (29). Briefly, cells of each bacterium were resuspended in ddH_2_O and the suspensions were used as templates. PCR was performed in RCR instrument (Bio-Gener, Hangzhou, China) with initial denaturation at 95°C for 2 min and 30 cycles of denaturation at 95°C for 30 s, annealing at 55°C for 30 s, extension at 72°C for 1 min, and a final extension step at 72°C for 10 min. The PCR products were sequenced by Youkang Biotechnology (Hangzhou, China), and the 16S rRNA sequences were aligned with known sequences in GenBank in NCBI using the BLAST. Phylogenetic analyses were implemented using the MEGA 11 software package, and phylogenetic trees were constructed using the neighbor-joining method (30).

### Inhibitory spectrum of *Glutamicibacter* sp. ZY1

The antagonistic assay was performed using the disk diffusion method (31). The tested bacteria included strains belonging to *Splendidus* clade, *Vibrio mediterranei, Vibrio atlanticus, Coralliilyticus* clade, *Orientalis* clade, *Photobacterium* sp., *Nereis* clade, *Harveyi* clade, *Anguillarum* clade, *Aeromonas hydrophila*, *Pseudomonas aeruginosa*, *Escherichia coli*, *Enterobacter bugandensis*, *Morganella morganii*, *Cholerae* clade, *Pseudoalteromonas* sp., *Halomonas* sp., and *Klebsiella variicola*. All these strains were grown in 2216E medium, except that *P. aeruginosa* and *E. coli* were separately cultured in LB medium. All bacterial strains were preserved in our laboratory. All the strains were cultured to optical density at 600 nm (OD_600_) of approximately 1.0 measured in a spectrophotometer (Allsheng, Hangzhou, China). Ten microliters of ZY1 was dropped on sterilized paper disks with a diameter of 0.6 cm and air dried. These filter paper disks were then put onto the bacterial lawn on the agar plates, and the plates were incubated at 28°C. After incubation overnight, the diameter of the inhibitory circle was measured. Each bacterium was independently cultured three times, and the antagonist assay was performed in triplicate for each tested bacterium.

### Inhibitory activity of ZY1 on *V. parahaemolyticus* YDE17

To determine the optimal inhibitory ratio of ZY1 on YDE17 *in vitro*, the inhibitory activity of ZY1 on YDE17 was determined using the disk diffusion method with different bacterial cell ratios as described previously (31). Briefly, YDE17 and ZY1 were separately cultured in 2216E liquid medium until the OD_600_ reached approximately 1.0. YDE17 was 10-fold serially diluted with 2216E liquid medium, and the cell densities of suspensions ranged from 1 × 10^1^ to 1 × 10^8^ CFU/mL. One hundred microliters of each YDE17 dilution was spread onto the 2216E plate. ZY1 was also 10-fold serially diluted with 2216E liquid medium to make suspensions from 1 × 10^1^ to 1 × 10^8^ CFU/mL. Ten microliters of ZY1 was dropped on the sterilized filter paper disks and air dried. These filter paper disks were placed on 2216E agar plates with different cells of YDE17, and the plates were incubated at 28°C. After overnight incubation, the diameters of inhibitory circles were measured. Each bacterium was cultured three times to perform the inhibitory assay with three independent measurements.

### Inhibition of YDE17 in seawater by ZY1

The inhibition of ZY1 on YDE17 in seawater was performed as described by Tang et al. (32). Briefly, ZY1 and YDE17 were separately cultured overnight until OD_600_ was 1.0. Four milliliters of ZY1 or YDE17 cultures was centrifuged at 12,000 rpm for 5 min, and the supernatant was filtered through a 0.22 µm pore size filter (Millipore, Darmstadt, Germany) to obtain two kinds of conditioned cell-free supernatant. Another 1.5-mL aliquot of the YDE17 culture was centrifuged at 12,000 rpm for 5 min, followed by resuspension of the cell pellet in 15 mL of sterilized seawater. The cell suspension of YDE17 was evenly divided into 24 aliquots, with 500 µL of each aliquot. The experimental groups were supplemented with 200 µL, 400 µL, 600 µL, and 800 µL of cell-free supernatant of ZY1, and the control groups were supplemented with 200 µL, 400 µL, 600 µL, and 800 µL of cell-free supernatant of YDE17, respectively. For each supplemented volume, three biological replicates were set. Finally, artificial sterilized seawater was added to make up to a total volume of 5 mL, and the 24 samples were incubated statically at 28°C for 24 h. To count the cell number of YDE17 that remained in the seawater, the samples were 10-fold serially diluted using artificial sterilized seawater, and 10 µL of each dilution was dropped on 2216E agar plates. The plates were incubated at 28°C for 24 h, and the single colonies that appeared in one appropriate drop were counted.

### Characterization of the inhibitory substance of ZY1

ZY1 and YDE17 were separately cultured overnight until OD_600_ reached 1.0. The culture of ZY1 was centrifuged at 12,000 rpm for 5 min, and the cell-free supernatant was obtained as described above. The cell-free supernatant of ZY1 was treated under different temperatures, proteinase K (Vazyme, Nanjing, China), and pHs, and the stability of the inhibitory substances under these conditions was then determined.

In order to determine the thermal stability of the inhibitory substances, cell-free supernatant of ZY1 was incubated at 40°C, 50°C, 60°C, 70°C, 80°C, 90°C, and 100°C for 1 h, respectively. The remaining inhibitory activity of cell-free supernatant of ZY1 was then determined at 28°C. To determine the effects of proteinase K on the inhibitory substances, cell-free supernatant of ZY1 was mixed with proteinase K at a ratio of 1:1 (vol/vol), incubated at 37°C for 1 h, then heated at 80°C for 10 min. The inhibitory activity of cell-free supernatant of ZY1 was then determined. To determine the pH stability of the inhibitory substances, the pHs of cell-free supernatant of ZY1 were subsequently adjusted to 4.0, 6.0, 8.0, 10.0, and 12.0 using 1 M HCl or 1 M NaOH and measured using a pH meter (Lei ci, Shanghai, China), respectively. After being incubated at different pHs for 1 h, the solution was adjusted back to the initial pH of 7.2 and was tested for the inhibitory activity. The bacterial lawn on the 2216E plate was prepared by mixing molten 2216E agar with 1% (vol/vol) culture of YDE17 at OD_600_ of 0.9 (33). For each experiment, independent replicates were performed for more than three times from the initial treatment under different conditions.

### Inner membrane permeability

Based on the method described previously (34, 35), the β-galactosidase leaks outside of the cell and degrades the colorless *o*-nitrophenyl-β-D-galactopyranoside (ONPG, Solarbio, Beijing, China) to produce yellow *o*-nitrophenol and galactose when the inner membrane becomes permeable. Therefore, in this study, we evaluated the effect of the cell-free supernatant of ZY1 on the permeability of the inner membrane of YDE17 by the addition of ONPG. YDE17 and ZY1 were separately cultured overnight at 28°C to an OD_600_ of approximately 1.0, and the cultures were centrifuged at 10,000 rpm for 10 min. Cell-free supernatant of ZY1 was obtained as described above. The cell pellet of YDE17 was resuspended in the artificial sterilized seawater to make a cell suspension with OD_600_ of approximately 0.4. For the experimental group, 2 mL of cell-free supernatant of ZY1, 2 mL of YDE17 bacterial suspension, and 200 µL of ONPG solution (stock solution, 5 mg/mL) were mixed together. Two control groups were set, i.e., 2 mL artificial sterilized seawater instead of cell-free supernatant of ZY1 or YDE17 bacterial suspension. The reaction mixtures were shaken at 28°C and absorbance, at 420 nm was measured at a time interval of 1 h. Data were obtained from three independent replicates that were performed from the independent culture growth.

### Measurement of siderophore level

The siderophore produced by bacteria was determined using the chrome azurol S (CAS) assay in both liquid medium and agar plate (36, 37). ZY1 was cultured in 2216E liquid medium at 28°C to different time intervals, and cell-free supernatant of ZY1 was obtained as described above. One milliliter of the cell-free supernatant from each culture, 1 mL of CAS bright blue dye, and 20 µL of shuttle solution were mixed. 2216E liquid medium, CAS dye, and shuttle solution were mixed and used as a control. The mixtures were incubated at room temperature for 5 min, followed by measurement of OD_630_ using a spectrophotometer. Quantitative determination of siderophore levels was calculated using the following equation: siderophore% = [(Ar − As)/Ar] × 100, where Ar = absorbance of reference and As = absorbance of sample. For the qualitative determination, 100 µL of the cell-free supernatants of ZY1 at different OD_600_ values was dropped on the solid CAS agar plates (Hopebio, Qingdao, China), respectively. The appearance of the orange-yellow circle at the drop site was defined to be obvious production of siderophores, and the diameter of the orange-yellow circle was positively correlated with the level of the siderophores (23). More than three independent cultures were used for three independent measurements to determine the siderophore production.

To compare the siderophores produced by YDE17 with or without the cell-free supernatant of ZY1, the cell-free supernatant of ZY1 was collected at two cell densities: one was OD_600_ ≈ 0.7 with no obvious siderophore production and the other was OD_600_ ≈ 0.9 with obvious siderophore production observed on the CAS plate. Culture of YDE17 at OD_600_ of 1.0 was collected. Three samples, (i) 100 µL of the cell-free supernatant of ZY1 mixed with 100 µL of 2216E medium, (ii) 100 µL of YDE17 mixed with 100 µL of 2216E medium, and (iii) 100 µL of the cell-free supernatant of ZY1 mixed with 100 µL of YDE17 suspension, were simultaneously dropped on CAS agar plate. The plates were incubated at 28°C for 16 h, and the diameters of the orange-yellow circle were measured. Replicates were performed for more than three times from three independent cultures and measurements.

### Determination of the type of siderophores produced by ZY1 and YDE17

Arnow’s test (38), FeCl_3_ experiment (39), and CAS test (40) were used to determine the types of siderophores produced by ZY1 and YDE17, respectively. YDE17 and ZY1 were separately cultured overnight at 28°C to an OD_600_ of approximately 1.0, and the cultures were centrifuged at 10,000 rpm for 10 min. Cell-free supernatants of ZY1 and YDE17 were obtained as described above. For Arnow’s test, 1 mL supernatant/medium, 1 mL of 0.5 M HCl, 1 mL nitrate molybdate reagent (10 g NaNO_2_ and 10 g Na_2_MoO_4_ dissolved in 100 mL ddH_2_O), and 1 mL of 1 M NaOH were mixed. The mixture was incubated at room temperature for 5 min, and the color change of the solution was observed to indicate the catecholate siderophore. For the FeCl_3_ experiment, 1 mL of the supernatant was subsequently supplemented with 1 or 5 mL of 2% FeCl_3_ (Aladdin, Shanghai, China). If the solution immediately turns red when 1 mL of 2% FeCl_3_ solution is added, it indicates that the supernatant contains the hydroxamate siderophore; if the supernatant only turns red or purple when 5 mL of 2% FeCl_3_ solution is added, it indicates that the supernatant contains the catecholate siderophore. For the CAS test, 200 µL cell culture of ZY1 and YDE17 was separately dropped on a CAS plate. If the color changes to purple, the bacterium contains the catecholate siderophore; if the color changes to orange, the bacterium contains α-hydroxycarboxylates, i.e., vibrioferrin for *V. parahaemolyticus* (41); if the color changes to yellowish, the bacterium secretes carboxylate siderophore.

### Effect of iron on inhibitory activity

To evaluate the effect of iron on inhibitory activity of cell-free supernatant of ZY1 on YDE17, cell-free supernatant of ZY1 with an OD_600_ of 0.9 was obtained as above. Cell-free supernatant of ZY1 was mixed with FeCl_3_ at various concentrations, i.e., 200 μM, 400 μM, 600 μM and 800 μM, at a ratio of 1:1 (vol/vol) as described by Yang et al. (23). YDE17 was cultured overnight until OD_600_ was about 1.0, the YDE17 lawn was prepared, and the inhibitory assay was performed as described previously (33); then, the diameter of the inhibitory circle was measured. Independent cultures of ZY1 and YDE17 were grown for three times and were used for the triplicate inhibitory experiments.

### Effect of iron on siderophore production

To determine whether iron level could affect the siderophore production, the change in color of YDE17 on a CAS agar plate was observed. Seven group samples were dropped on the CAS plates: (i) 100 µL of the cell-free supernatant of ZY1 mixed with 100 µL of 2216E medium, (ii) 100 µL of YDE17 mixed with 100 µL of 2216E medium, (iii) 100 µL of the cell-free supernatant of ZY1 mixed with 100 µL of YDE17, (iv–vii) 100 µL of YDE17 supplemented with 100 µL of the cell-free supernatant of ZY1 and FeCl_3_ at levels of 200 µM, 400 µM, 600 µM, and 800 µM, respectively. The seven samples were simultaneously dropped on CAS plates, and the diameter of the orange-yellow circle was measured. Independent cultures of ZY1 and YDE17 were separately grown for three times and were used for the triplicate experiments.

### Artificial infections

Healthy Pacific white shrimp *L. vannamei* (mean length, 1 ± 0.03 cm) were purchased from the Mei Tai shrimp farm in Zhuhai, Guangzhou. They were cultured at 27°C for 7 days with air inflation, then the shrimps were challenged by different bacterial combinations using immersion infection mode. The shrimps were fed with basic feed four times a day. To calculate the mean lethal doses (LD_50_) of YDE17, 1,350 shrimps were divided into 27 groups with 50 shrimps in each groupShrimps were infected with YDE17 at concentrations of 5 × 10⁴ CFU/mL, 1.25 × 10⁵ CFU/mL, 2.5 × 10⁵ CFU/mL, 5 × 10⁵ CFU/mL, 1.25 × 10⁶ CFU/mL, 6.25 × 10⁶ CFU/mL, 3.125 × 10⁷ CFU/mL, and 1.5 × 10⁸ CFU/mL, while the control group was supplemented with the same volume of sterilized 2216E medium. For each bacterial concentration, three groups of shrimps were parallelly infected. The health status of shrimps was observed every 8 h for 5 days, and the number of dead shrimps was recorded. The LD_50_ of YDE17 were calculated using the Karber method (42). There were a few deaths of shrimps challenged by 1 × 10^5^ CFU/mL ZY1 in the preliminary experiment; thus, ZY1 at this concentration was used in the protective test. To demonstrate whether ZY1 exerts a protective effect, 600 shrimps were randomly divided into 12 groups with 50 shrimps in each group. The groups were separately challenged with YDE17 (1 × 10^6^ CFU/mL), ZY1 (1 × 10^5^ CFU/mL), ZY1 (1 × 10^5^ CFU/mL)/YDE17 (1 × 10^6^ CFU/mL), and a control group without bacterial inoculation. For each immersion infection, three replicates were performed. The clinical symptoms of individuals were observed, and the number of dead shrimps in each group was recorded. To further determine the effect of FeCl_3_ on the inhibitory effect of ZY1 *in vivo,* 800 µM FeCl_3_ and 40 µM 2,2-bipyridyl (DP, iron chelator, Macklin, Shanghai, China) were also used in the subsequent infection experiment. Seven hundred fifty shrimps were randomly divided into 15 groups with 50 shrimps in each group. The groups were YDE17 (1 × 10^6^ CFU/mL)/ZY1 (1 × 10^5^ CFU/mL)/FeCl_3_, YDE17 (1 × 10^6^ CFU/mL)/DP, FeCl_3_, DP and a control group without any treatment. Three replicates were performed for each group. Individuals were observed for clinical signs, and the number of dead shrimps in each group was recorded. The following formula was used to calculate survival rate (%) and relative percent survival (RPS, %) (43) :


$$survival\;rate\;\left(\%\right)\;=\;100\;\times \;\frac{Survival\;number\;of\;each\;treatment\;after\;challenge}{\;Total\;number\;before\;challenge};$$

$$RPS\;\left(\%\right)\;=\;100\;\times \;\left(1-\frac{Mortality\;rate\;of\;the\;co-infected\;group}{Mortality\;rate\;of\;YDE17\;group}\right).$$

### Statistical analysis

All data were obtained in triplicate with similar results. Statistical analyses were carried out using GraphPad Prism (GraphPad Software). The two-tailed unpaired Student’s *t*-test was used to analyze all data, except for the determination of survival rate. Data were presented as mean ± standard deviation (SD). In all cases, differences were defined as *, *P* < 0.05; **, *P* < 0.01; ***, *P* < 0.001, and ****, *P* < 0.0001.

## RESULTS

### Screening, isolation, and characterization of antagonistic bacteria

**Fig 1:**
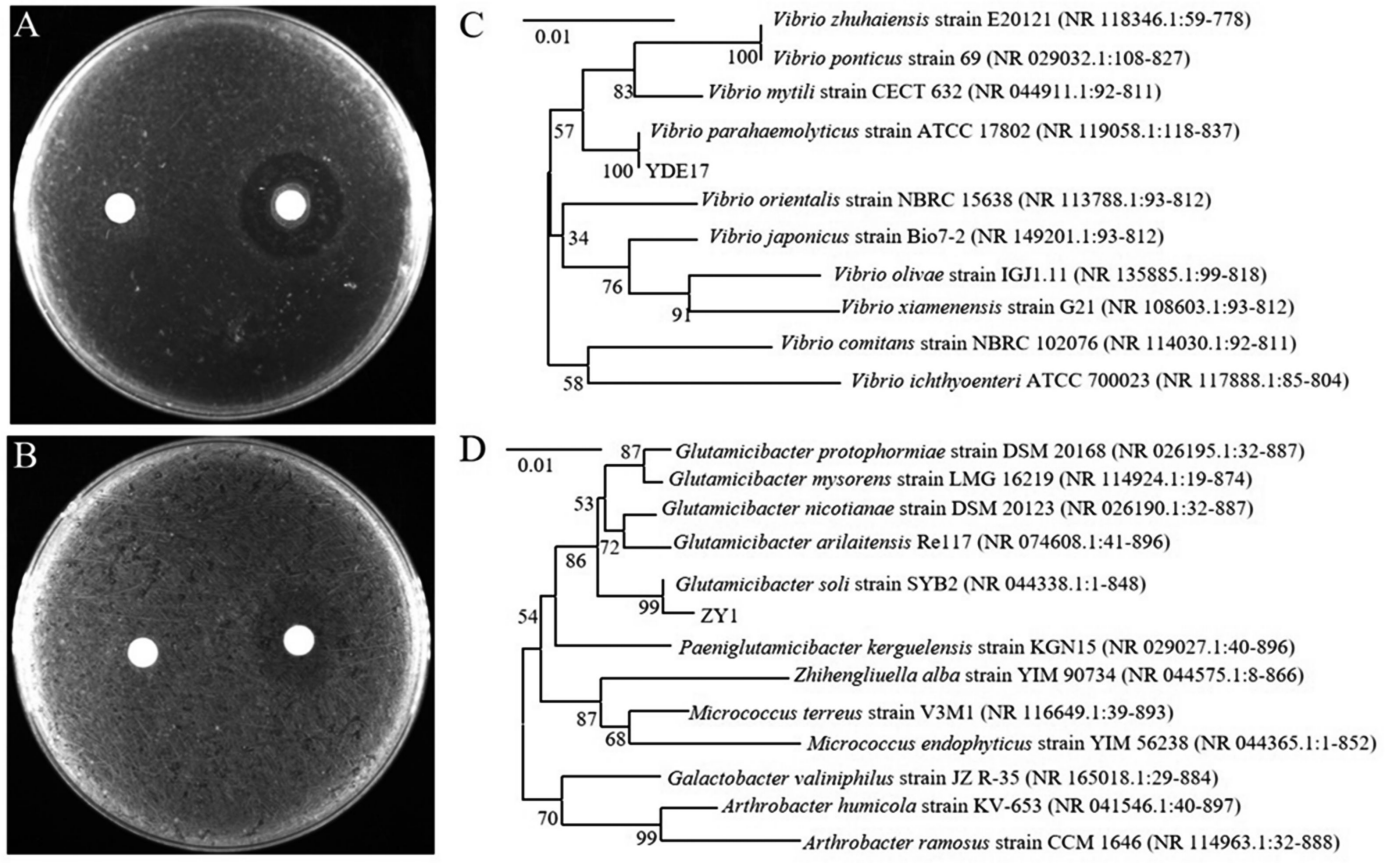
Bacterial isolation and identification. (**A**) Strain ZY1 antagonized YDE17. Fifty microliters of YDE17 was spread on 2216E plate, with the filter disk dipped with 2216E (left) and filter disk dipped with ZY1 cultured in 2216E medium (right), and the plate was incubated at 28°C for 24 h. (**B**) Cell-free supernatant of ZY1 antagonized YDE17. Fifty microliters of YDE17 was spread on 2216E plate, with the filter disk dipped with 2216E (left) and filter disk dipped with cell-free supernatant of ZY1 cultured in 2216E medium (right), and the plate was incubated at 28°C for 24 h. (**C**) Phylogenetic tree of YDE17 constructed using 16S rRNA. (**D**) Phylogenetic tree of ZY1 constructed using 16S rRNA.

In the water samples collected from the shrimp farm, 21 species of culturable strains were isolated and identified (Table S1). Among them, the inhibitory effect of ZY1 on YDE17 was demonstrated with the largest inhibition zone (Fig. 1A). Furthermore, the cell-free supernatant of ZY1 had the same antagonistic effect as the intact cells (Fig. 1B). The 16S rRNA gene sequence of YDE17 and subsequent phylogenetic analysis indicated that YDE17 was *V. parahaemolyticus*, with homology to the *V. parahaemolyticus* strain ATCC 17802 (Fig. 1C). The 16S rRNA gene sequence of ZY1 and subsequent phylogenetic analysis indicated that ZY1 belonged to the *Glutamicibacter soli*, which is homologous to the *G. soli* strain SYB2 (Fig. 1D).

### Inhibitory spectrum of *Glutamicibacter* sp. ZY1

To determine the inhibitory spectrum of ZY1, 28 different species of bacteria were used to perform the inhibitory assay on 2216E or LB agar plates. ZY1 antagonized many kinds of bacteria, including the *Splendidus* clade, *Coralliilyticus* clade, *Orientalis* clade, *Photobacterium* sp., *Harveyi* clade, *Anguillarum* clade, and *Pseudoalteromonas* sp., but could not antagonize the *Nereis* clade, *Cholerae* clade, *V. atlanticus*, *A. hydrophila*, *P. aeruginosa*, *E. coli*, *E. bugandensis*, *M. morganii*, *Halomonas* sp., and *K. variicola* strains (Table 1; Fig. S1).

**TABLE 1:** Strains used to determine the inhibitory spectrum of ZY1^*a*^

Strain name	Clade	Genus/species name (with high similarity)	Size of the antagonistic zone/cm (mean ± SD)
AJ01	Splendidus	*Vibrio splendidus*	2.2 ± 0.1
S1		*V*. *mediterranei*	1.3 ± 0.3
S2	Splendidus	*V*. *crassostreae*; *V*. *coralliirubri*; *V*. *gigantis*	2.87 ± 0.07
S7		*V*. *atlanticus*; *V*. *cyclitrophicus*; *V*. *tasmaniensis*	0.6
S8	Splendidus	*V*. *crassostreae*; *V*. *pomeroyi*; *V*. *cyclitrophicus*	2.03 ± 0.03
Y10n	Splendidus	*V. pelagius*; *V*. *fortis*; *V*. *xiamenensis*	2.73 ± 0.12
SPS2	Coralliilyticus	*V*. *coralliilyticus*	2.33 ± 0.03
SPS11	Orientalis	*V*. *brasiliensis*	2.40 ± 0.10
PTS6		*Photobacterium ganghwense*	1.97 ± 0.04
Y1n	Nereis	*V*. *nereis*; *V*. *hepatarius*; *V*. *coralliilyticus*	0.6
Y3n	Nereis	*V*. *nereis*	0.6
G2	Harveyi	*V. alginolyticus*; *V. owensii*; *V. hyugaensis*	2.4 ± 0.1
H1	Harveyi	*V*. *alginolyticus*	1.4 ± 0
Y9n-1	Harveyi	*V*. *natriegens*; *V*. *alginolyticus*	1.33 ± 0.07
G3n	Harveyi	*V*. *rotiferianus*	2.43 ± 0.04
Sps1	Harveyi	*V*. *parahaemolyticus*	1.37 ± 0.12
W3	Harveyi	*V*. *jasicida*; *V*. *owensii*; *V*. *rotiferianus*	2.00 ± 0.01
Va01	Anguillarum	*V*. *anguillarum*	2.93 ± 0.04
YD17		*Aeromonas hydrophila*	0.6
PA		*Pseudomonas aeruginosa*	0.6
DH5α		*Escherichia coli*	0.6
YD6		*Enterobacter bugandensis*	0.6
YD9		*Morganella morganii*	0.6
YDE13	Cholerae	*V*. *cholerae*	0.6
H2		*Pseudoalteromonas* sp.	2.06 ± 0.04
NTS6		*Halomonas* sp.	0.6
YH4		*Klebsiella variicola*	0.6

^*a*^ The size of the inhibitory circle included the diameter of the filter disk (0.6 cm).

### Inhibitory effect of ZY1 on the growth of YDE17 in different matrices

**Fig 2:**
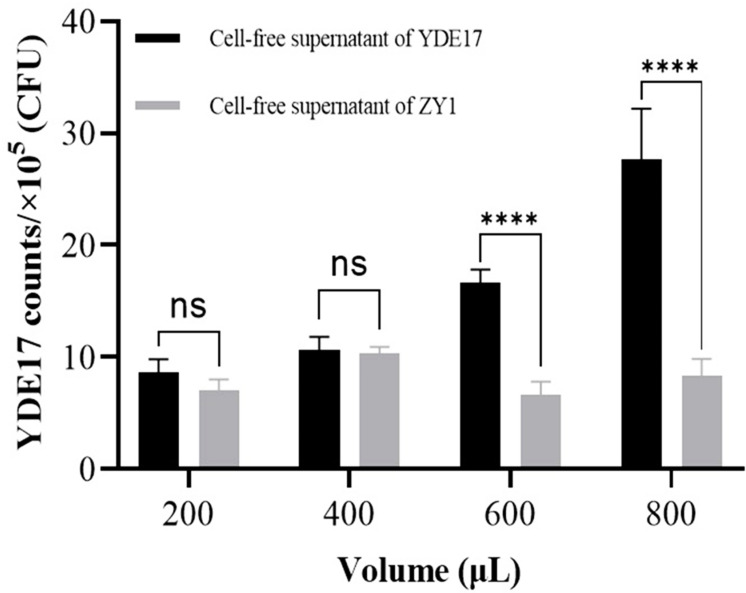
Inhibition of YDE17 by the cell-free supernatant of ZY1 in artificial seawater. Different volumes of the cell-free supernatant of ZY1 were supplemented into the YDE17 suspension in seawater, and the cell-free supernatant of YDE17 itself was also supplemented as a control. For each addition volume, three biological replicates were performed. The 24 samples were incubated at 28°C for 24 h, and the cells of remaining YDE17 in the artificial seawater were numbered using the dropped plate method. Data are mean values ± SD. ****, *P* < 0.0001.

The inhibitory effect of ZY1 on the growth of YDE17 was in a dose-dependent manner. When more ZY1 co-incubated with less YDE17, it showed the larger inhibitory circle. In detail, the cells of YDE17 below 1 × 10^4^ CFU could not be grown to lawns, while even if there were as few as 1 × 10^3^ CFU bacterial cells of ZY1, it exhibited inhibitory activity on the thinnest YDE17 lawn grown from the 1 × 10^4^ CFU. The inhibitory effect of ZY1 could steadily be detected when the cell numbers were more than 1 × 10^5^ CFU, regardless of the level of YDE17 on the agar plates. The largest inhibitory circle appeared when the cell number of ZY1 was 1 × 10^8^ CFU and the cell number of YDE17 was 1 × 10^4^ CFU (Table 2; Fig. S2). Furthermore, to test whether this inhibition could occur in the real seawater sample, the cell-free supernatant of ZY1 was applied to YDE17 suspension in artificial seawater. As the volume of the cell-free supernatant of ZY1 increased, the number of YDE17 cells gradually decreased, indicating that the cell-free supernatant of ZY1 could inhibit YDE17 cells. When the volume of the cell-free supernatant of ZY1 reached 800 µL, the cell number of YDE17 was only 8 × 10^5^ CFU, but the cell number of YDE17 in the control group was approximately 2.8 × 10^6^ CFU (Fig. 2), which indicated that the growth of *V. parahaemolyticus* YDE17 could be inhibited by approximately 72% in the presence of the cell-free supernatant of ZY1.

**TABLE 2:** Diameters of the inhibitory circle of ZY1 on the YDE17 lawn at different cell ratios^*c*^

ZY1^*b*^ ratio	Diameter at YDE17*^a^* ratio of:							
	10^1^	10^2^	10^3^	10^4^	10^5^	10^6^	10^7^	10^8^
10^1^	0.6	0.6	0.6	0.6	0.6	0.6	0.6	0.6
10^2^	0.6	0.6	0.6	0.6	0.6	0.6	0.6	0.6
10^3^	0.6	0.6	0.6	1 ± 0.01	0.6	0.6	0.6	0.6
10^4^	0.6	0.6	0.6	1.1 ± 0.01	0.6	0.6	0.6	0.6
10^5^	0.6	0.6	0.6	2 ± 0.02	2 ± 0.01	0.9 ± 0.01	1 ± 0.02	0.9 ± 0.01
10^6^	0.6	0.6	0.6	2.3 ± 0.02	2 ± 0.01	1.4 ± 0.01	1.4 ± 0.01	1.4 ± 0.01
10^7^	0.6	0.6	0.6	3.1 ± 0.01	2.5 ± 0.03	1.9 ± 0.02	1.8 ± 0.02	1.7 ± 0.02
10^8^	0.6	0.6	0.6	3.6 ± 0.01	2.7 ± 0.02	2.2 ± 0.01	1.9 ± 0.02	1.9 ± 0.03

^*a*^ The cell density of YDE17 suspension (indicated in the first row) and 100 μL of each dilution were spread on each plate.

^*b*^ The cell density of ZY1 suspension (indicated in the first column) and 10 μL of each dilution were dropped onto the filter disk.

^*c*^ The diameters of the inhibitory circle included the diameter of the filter disk, which is 0.6 cm.

### Characterization of the inhibitory effect of ZY1

**Fig 3:**
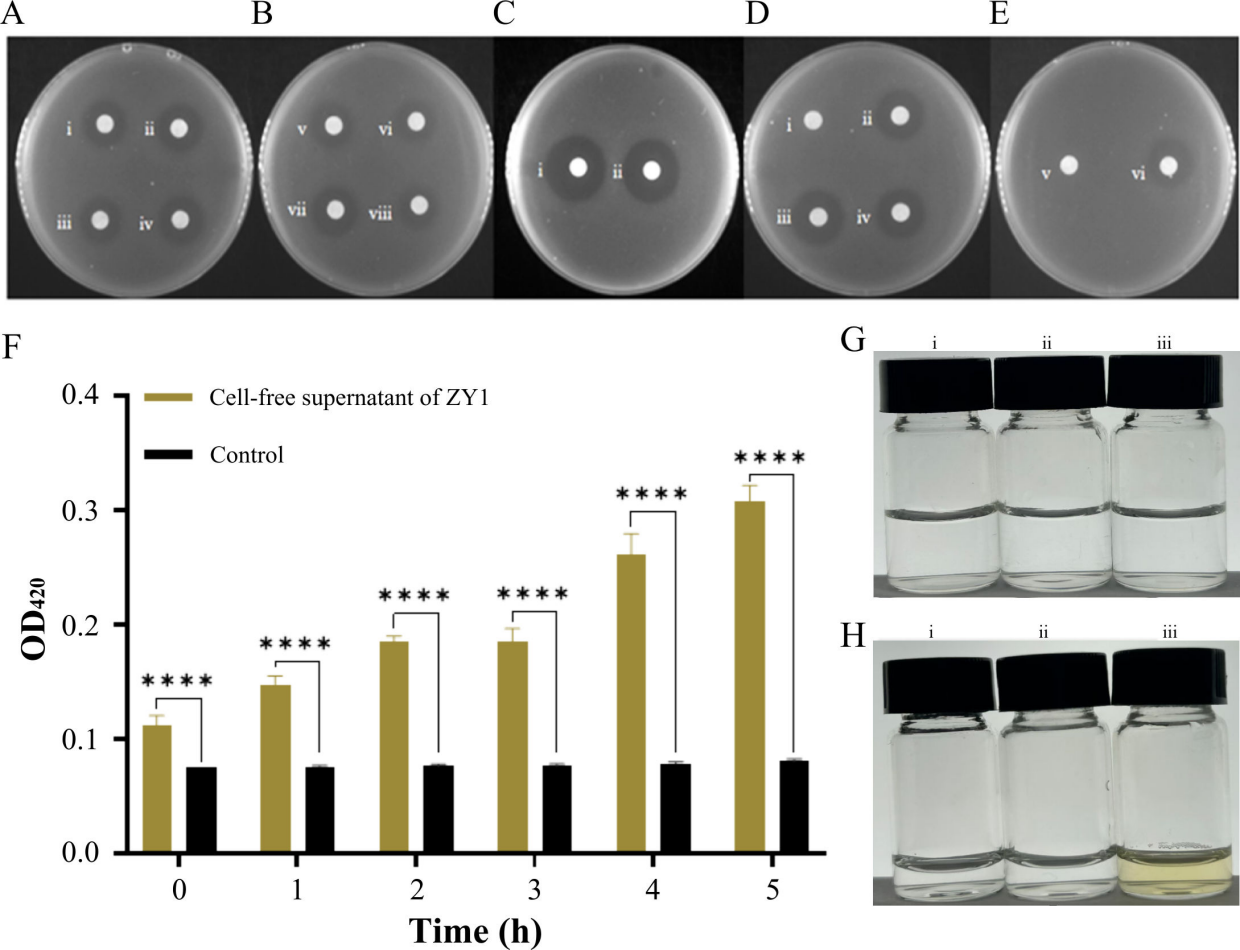
Characterization of the inhibitory activity of the cell-free supernatant of ZY1. (**A and B**) The inhibitory activity of the cell-free supernatant of ZY1 treated at different temperatures. The numbers “i, ii, iii, iv, v, vi, and vii” represented the cell-free supernatant of ZY1 treated under 40°C, 50°C, 60°C, 70°C, 80°C, 90°C, and 100°C, respectively, the number “viii” represented the cell-free supernatant of ZY1 without treatment. (**C**) The inhibitory activity of the cell-free supernatant of ZY1 treated with proteinase K. The number “i” represented the cell-free supernatant of ZY1 without treatment, “ii” represented the cell-free supernatant of ZY1 treated with proteinase K. (**D and E**) The inhibitory activity of the cell-free supernatant of ZY1 treated under different pHs. The numbers “i, ii, iii, iv, and v” represented pH = 4, 6, 8, 10, and 12, respectively. The number “vi” represented the cell-free supernatant of ZY1 without treatment. (**F**) Inner membrane permeability of YDE17 treated with the cell-free supernatant of ZY1; control: 2 mL of artificial seawater mixed with 2 mL of YDE17 and 200 µL of ONPG; cell-free supernatant of ZY1: 2 mL of the cell-free supernatant of ZY1, 2 mL of YDE17, and 200 µL of ONPG. (**G**) Color of the samples before reaction. (**H**) Color of the samples after the reaction for 3 h. (i) 2 mL of cell-free supernatant of ZY1 mixed with 2 mL of seawater and 200 µL of ONPG; (ii) 2 mL of YDE17 mixed with 2 mL of artificial seawater and 200 µL of ONPG; (iii) 2 mL of cell-free supernatant of ZY1, 2 mL of YDE17 and 200 µL of ONPG. All the measurements were performed in triplicate from three independent cultures. Data are mean values ± SD. ****, *P* < 0.0001.

Since the cell-free supernatant of the ZY1 culture had an inhibitory effect, it was used to characterize the inhibitory activity under different treatments. After treatment under different temperatures, the inhibitory activity of the cell-free supernatant of ZY1 did not change (Fig. 3A and B). To determine the effect of proteinase K, the inhibitory activity of the cell-free supernatant of ZY1 with and without proteinase K treatment was compared. The results showed that the inhibitory activity of the cell-free supernatant of ZY1 hardly changed after treatment with proteinase K (Fig. 3C). After treatment under different pHs ranging from 6 to 10, the inhibitory activity remained, but extremely acidic and alkaline conditions decreased the inhibitory activity of the cell-free supernatant of ZY1 (Fig. 3D and E).

β-galactosidase result showed that the absorbance of YDE17 co-incubated with the cell-free supernatant of ZY1 increased, but the absorbance of the control group barely changed (Fig. 3F). The samples were colorless before the reaction, but after incubation for 3 h, the color of the reaction mixture with YDE17 and the cell-free supernatant of ZY1 changed to yellow; however, the two control groups remained colorless (Fig. 3G and H). These results indicated that the cell-free supernatant of ZY1 could damage the inner membrane of YDE17 cells.

In summary, the inhibitory activity of the cell-free supernatant of ZY1 exhibited the following characteristics: thermal stability, proteinase K stability, and pH stability within the range of 6–10. These characteristics led us to speculate that the inhibitory substances in the cell-free supernatant of ZY1 were small and nonprotein molecules. Since iron competition is the strategy usually occupied by probiotics, we further wonder whether the siderophores produced by ZY1 were the functional inhibitory substances on YDE17.

### ZY1 inhibited YDE17 through siderophore production to compete for iron

**Fig 4:**
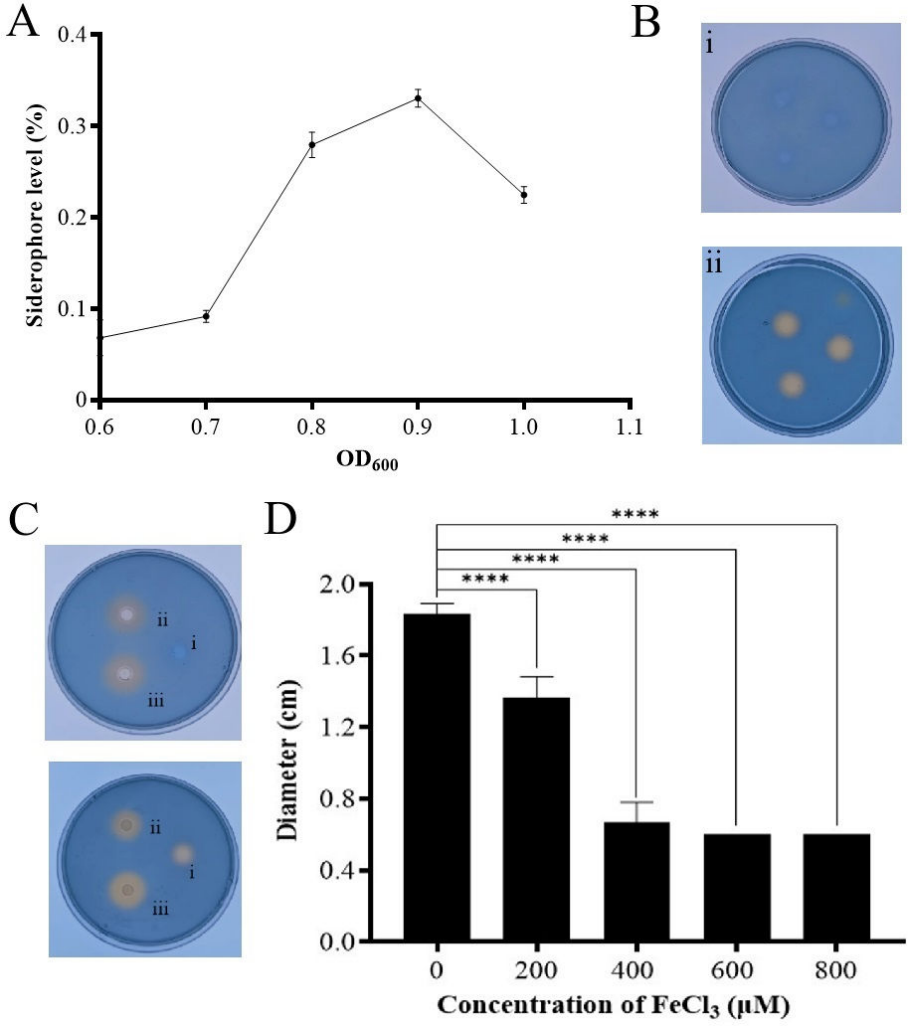
Siderophore productions of ZY1 and YED17 under different conditions. (**A**) Siderophore levels of ZY1 at different OD_600_ values. (**B**) The cell-free supernatant of ZY1 at different OD_600_ dropped on CAS plates: (i) the three replicates of cell-free supernatant of ZY1 at an OD_600_ of 0.4 dropped on a CAS plate; (ii) the three replicates of cell-free supernatant of ZY1 at an OD_600_ of 0.9 dropped on a CAS plate. (**C**) The effect of cell-free supernatant of ZY1 on the siderophore production of YDE17. The cell-free supernatant was obtained from ZY1 at an OD_600_ of 0.7 (upper): (i) the cell-free supernatant of ZY1, (ii) YDE17 suspension, and (iii) the cell suspension of YDE17 mixed with the cell-free supernatant of ZY1 dropped on a CAS plate. (C) The cell-free supernatant was obtained from ZY1 at an OD_600_ of 0.9 (lower): (i) the cell-free supernatant of ZY1, (ii) YDE17 suspension, and (iii) the cell suspension of YDE17 mixed with the cell-free supernatant of ZY1 dropped on a CAS plate. (D) Diameters of the inhibitory circle generated by cell-free supernatant of ZY1 with different concentrations of FeCl_3_. The diameter of the inhibitory circle included the diameter of the filter paper sheet (0.6 cm). The data are the mean values of three replicates ± SD. ****, *P* < 0.0001.

The siderophore level in the supernatant of ZY1 was cell density dependent, with a trend of first increasing and then decreasing. When ZY1 was cultured to an OD_600_≈0.9, the siderophore level reached a maximum level, then, the level of siderophore decreased. When the OD_600_ of ZY1 was less than 0.7, the level of siderophores was lower (Fig. 4A). CAS assays showed that the color on the CAS plate did not change when the cell-free supernatant of ZY1 at OD_600_ ≈0.4 was dropped (Fig. 4Bi); however, the color on the CAS plate changed to orange-yellow when the cell-free supernatant of ZY1 at OD_600_ ≈0.9 was dropped (Fig. 4Bii). Taken all the results together, it can be deduced that the inhibition of ZY1 on YDE17 might be attributed to the siderophores produced by ZY1, which created an iron-limiting condition for YDE17. To verify this postulation, the siderophore production of YDE17 in the presence of the cell-free supernatant of ZY1 was determined. The results showed that when the cell-free supernatant of ZY1 at an OD_600_ ≈ 0.7 with no color change was supplemented, the orange-yellow circle of YDE17 was larger than that without the cell-free supernatant. The similar phenomenon was also observed when the cell-free supernatant of ZY1 at an OD_600_ ≈ 0.9 was collected and applied (Fig. 4C). Therefore, the presence of the cell-free supernatant of ZY1 likely induced the siderophore secretion of YDE17, which could also support the incomplete inhibition of YDE17 growth by ZY1 vice versa.

To further confirm that the inhibitory effect of ZY1 was exerted by iron competition, the inhibitory assay was performed with FeCl_3_ supplementation. FeCl_3_ concentrations of 200 µM, 400 µM, 600 µM, and 800 µM were tested, and none of which affected the growth of YDE17 (Fig. S3A). As the levels of FeCl_3_ in the cell-free supernatant of ZY1 increased, the inhibitory circle decreased. When the FeCl_3_ concentration increased to 800 µM, the inhibitory circle completely disappeared (Fig. 4D; Fig. S3B through F). Thus, the inhibitory effect of ZY1 on the growth of YDE17 was mainly attributed to iron competition.

### ZY1 produced hydroxamate and α-hydroxycarboxylate siderophores, while YDE17 produced vibrioferrin

**Fig 5:**
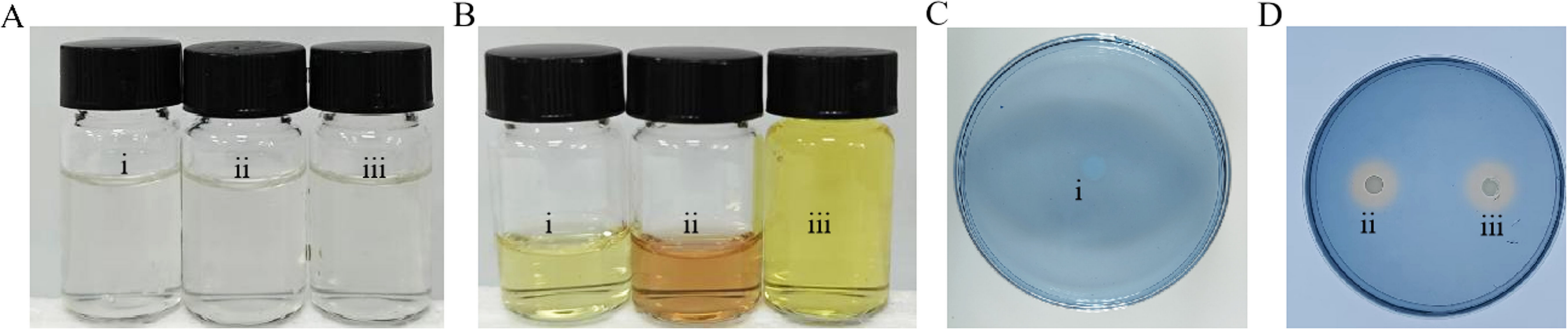
Determination of the siderophore production of ZY1 and YDE17. (**A**) Anow’s test: (i) the control 2216E medium; (ii) cell-free supernatant of ZY1; (iii) cell-free supernatant of YDE17. (**B**) FeCl_3_ test: (i) the control 2216E medium; (ii) cell-free supernatant of ZY1; (iii) cell-free supernatant of YDE17. (**C and D**) CAS test: (i) the control 2216E medium; (ii) cell culture of ZY1; (iii) cell culture of YDE17.

Because the siderophores played important roles in the competition between the two bacteria, the types of siderophores produced by both strains were further determined. In the Anow’s test, the color of the solution did not change to red after the addition of cell-free supernatant ZY1 or cell-free supernatant YDE17, indicating that the siderophore produced by both strains was not the catecholate type (Fig. 5A). In the FeCl_3_ test, the color of the solution changed to red when 1 mL of FeCl_3_ was added to cell-free supernatant of ZY1 (Fig. 5Bii), indicating that ZY1 produced the hydroxamate siderophore, but there was no change in the color whether 1 mL or 5 mL FeCl_3_ was added into the supernatant of YDE17 (Fig. 5Biii), indicating that YDE17 did not produce the hydroxamate siderophore or the catecholate siderophore. In the CAS experiment, the color of the CAS plate changed from blue to orange, indicating that the ZY1 secreted the α-hydroxycarboxylate siderophore and YDE17 secreted vibrioferrin (Fig. 5C and D).

**Fig 6:**
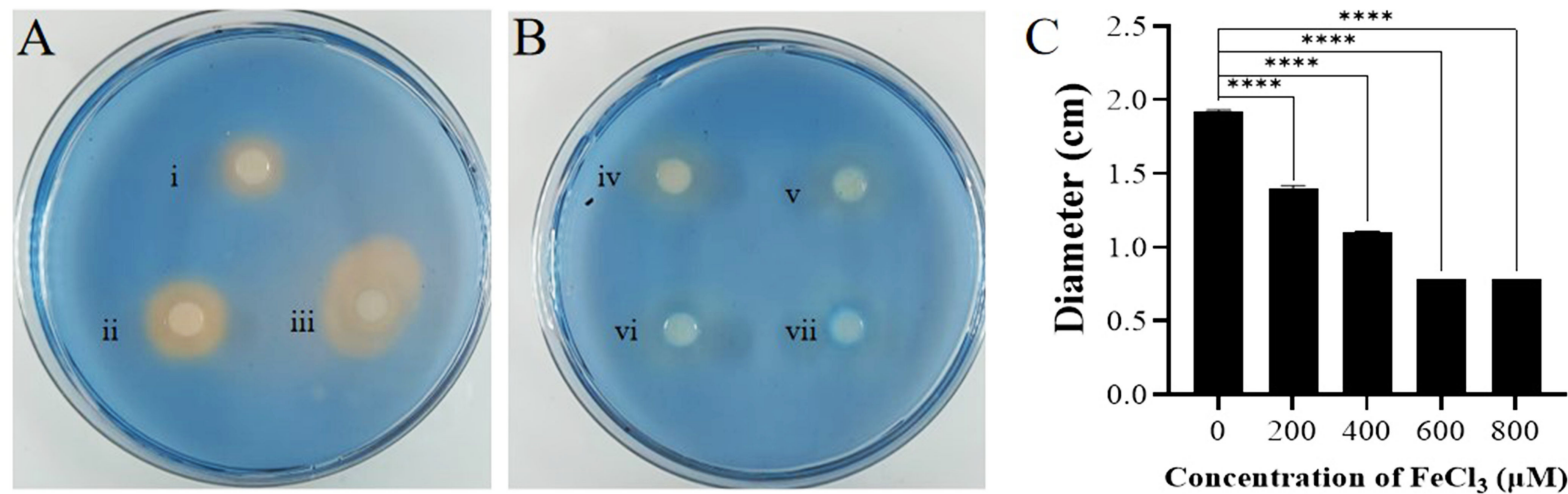
Effects of the cell-free supernatant of ZY1 on the siderophore production of YDE17 with different concentrations of FeCl_3_. (**A**) The siderophore productions of (i) cell-free supernatant of ZY1; (ii) YDE17 cells; (iii) YDE17 cells with the cell-free supernatant of ZY1. (**B**) The siderophore productions of (iv) YDE17 cells with the cell-free supernatant of ZY1 and 200 µM of FeCl_3_; (v) YDE17 cells with the cell-free supernatant of ZY1 and 400 µM of FeCl_3_; (vi) YDE17 cells with the cell-free supernatant of ZY1 and 600 µM of FeCl_3_; (vii) YDE17 cells with the cell-free supernatant of ZY1 and 800 µM of FeCl_3_. (**C**) Diameter of the orange-yellow color of (iii–vii) in the above figures. The diameter of the orange-yellow color included the diameter of the filter disk, which was 0.6 cm. All the measurements were performed in triplicate from three independent cultures. The data are presented as the means ± SD. ****, *P* < 0.0001.

The siderophore production of both strains in the presence of iron was further determined. FeCl_3_ at concentrations of 200 µM, 400 µM, 600 µM, and 800 µM was used. Then, cell-free supernatant of ZY1 with or without FeCl_3_ was applied to YDE17, and the siderophore production was detected. The results showed that as the FeCl_3_ concentration in the cell-free supernatant of ZY1 increased, the orange-yellow circle of YDE17 decreased, and the orange-yellow circle nearly disappeared when the concentration of FeCl_3_ was 800 µM (Fig. 6).

### ZY1 protected shrimp from YDE17 infection

**Fig 7:**
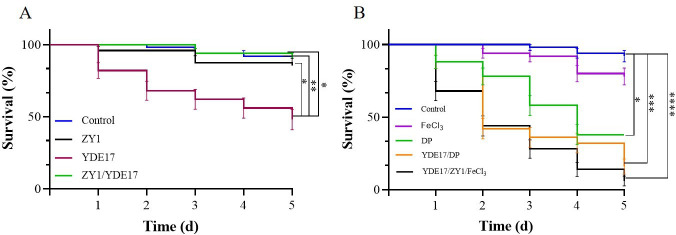
Survival rates of the different groups of shrimps. (**A**) The control group, the shrimp in seawater supplemented with 2216E medium; the YDE17 group, the shrimp challenged with 10^6^ CFU/mL YDE17; the ZY1 group, the shrimp challenged with 10^5^ CFU/mL ZY1; and the ZY1/YDE17 group, the shrimp simultaneously challenged with 10^6^ CFU/mL YDE17 and 10^5^ CFU/mL ZY1. All the groups were observed for 5 days, and the number of dead individuals was recorded. (**B**) The effect of iron on the infection of *V. parahaemolyticus in vivo*. The control group, the shrimp in water supplemented with 2216E medium; the FeCl_3_ group, the shrimp challenged with 800 µM FeCl_3_; the DP group, the shrimp challenged with 40 µM DP; the YDE17/DP group, the shrimp challenged with 10^6^ CFU/mL YDE17 and 40 µM DP; the YDE17/ZY1/FeCl_3_ group, the shrimp challenged with 10^6^ CFU/mL YDE17, 10^5^ CFU/mL ZY1, and 800 µM FeCl_3_. Shrimps in all the groups were observed for 5 days, and the number of dead individuals was recorded. The data are presented as the means ± SD. *, *P* < 0.05; **, *P* < 0.01; ***, *P* < 0.001; ****, *P* < 0.0001.

When challenged by different concentrations of YDE17, the survival rate of shrimp *L. vannamei* decreased as concentrations of YDE17 increased (Table 3). The infected shrimp showed signs of jejunum followed by whitening of the entire shrimp body and death. On the basis of these results, the LD_50_ of YDE17 was calculated as 1 × 10^6^ CFU/mL in the artificial immerse infection mode. In the group infected with ZY1 alone, the average survival rate of the shrimp was 86%, which indicated that ZY1 was relatively safe for shrimp. In the ZY1/YDE17 group, the shrimp started to die on the 3rd day, and the survival rate reached 90% on the 5th day. In the group infected with YDE17 alone, the survival rate of the shrimp was 40% (Fig. 7A). On the basis of these data, the RPS of ZY1 was calculated as 83.3%. Thus, ZY1 could protect shrimp from YDE17 infection.

**TABLE 3:** Survival of shrimp *Litopenaeus vannamei* challenged by different levels of YDE17

Concentration of YDE17 (CFU/mL)	Survival rate (%)
5.00 × 10^4^	100
1.25 × 10^5^	74
2.50 × 10^5^	72
5.00 × 10^5^	62
1.25 × 10^6^	0
6.25 × 10^6^	0
3.13 × 10^7^	0
1.50 × 10^8^	0

To determine the effect of FeCl_3_ on the inhibitory activity of ZY1 *in vivo*, the infection with different bacterial strains under different iron conditions was performed. FeCl_3_ showed a minor effect on the survival rates, but the shrimps treated with DP at the level without affection of growth of YDE17 (data not shown) showed a survival rate of 38%, which indicated that iron is important for the survival of shrimps. The survival rate of shrimp challenged with YDE17/ZY1/FeCl_3_ was 6%, which was significantly lower than 90% challenged with the co-infection of ZY1/YDE17 (Fig. 7B). This result indicated that the protective effect of ZY1 on YDE17 infection was regulated by iron level, and no protective effect was detected at higher iron levels in the *in vivo* immerse infection model.

## DISCUSSION

Actinomycetes are considered producers of a broad spectrum of antagonistic compounds or extracellular enzymes, and they are considered ideal probiotic candidates in aquaculture (44–46). Previously, the potential actinobacterial genera were limited to *Streptomyces*, *Micromonospora*, and *Salinispora* (45). The genus *Glutamicibacter* is an actinobacteria belonging to the family Micrococcaceae (47), and it is known for their production of antibiotics and enzymes that play crucial roles in the treatment of chronic human diseases (48). Studies have shown that *Glutamicibacter uratoxydans* KIBGEIB41 produces chitinase that hydrolyzes the cell wall of the fungus, increasing the resistance of the organism to the fungus (49). The *Glutamicibacter bergerei* 04–279 strain inhibits fish pathogens such as *V. anguillarum* and *Photobacterium damselae* (50). However, only a few studies have explored the antagonistic effects of *Glutamicibacter* sp. and active substances produced by *Glutamicibacter* sp. (51). Our study is the first to identify a *Glutamicibacter* sp. with the capacity to inhibit a diverse of pathogenic *Vibrio* spp., including a *V. parahaemolyticus* isolate that was highly virulent to shrimp. ZY1 exhibited a broad spectrum of inhibitory effects on *Vibrio* spp., and it exhibited an average diameter of 2.15 cm. The inhibitory ability of ZY1 was positively correlated with cell number, which was similar to the cell density-dependent inhibitory effects of *Lactiplantibacillus plantarum* and *Pediococcus acidilactici* on *Vibrio* spp. (52).

The role of siderophores in improving antimicrobial properties has become a popular research topic in recent years (20). For example, *Streptomyces* can inhibit the growth of *Vibrio* sp. by producing siderophores (53). In bacteria belonging to *Glutamicibacter* sp., the antimicrobial substances kinetin-9-ribose and embinin, produced by *Glutamicibacter mysorens* (48), and unknown molecules, produced by *Glutamicibacter* sp. FBE-19 (54), have been reported, but there have been no reports on the inhibition of pathogens via iron competition using siderophore production. The thermal stability, pH, and proteinase K stability of the inhibitory substrates indicated that the inhibitory substances were small and nonprotein molecules, similar to previous findings in *Streptomyces* sp. S073 and *Bacillus subtilis* strain O-741 (23, 55). With the attenuated inhibitory effect in the presence of increased iron levels, we postulated that the inhibitory substances produced by ZY1 were siderophores, through which ZY1 inhibited YDE17 via iron competition. However, unlike the complete inhibitory effects of *Streptomyces* (23), *Bacillus* sp. JK08 (56), and LAB (57) on *Vibrio* sp., the inhibitory effect of ZY1 was incomplete, which indicated that the inhibitory effect of ZY1 could induce a quick response of YDE17. The greater amount of siderophores produced by YDE17 in the presence of the cell-free supernatant of ZY1 further confirmed that YDE17 could produce more siderophores under the iron-limiting conditions created by the cell-free supernatant of ZY1, allowing the remaining bacterial cells to survive. This finding is consistent with the common knowledge that bacteria have the capacity to produce more siderophores under the iron-limiting condition created by iron chelator 2,2-bipyridyl (58). Depending on the chemical nature of the ligand that provides oxygen for Fe(III) coordination, siderophores can be classified into catecholates, hydroxamates, carboxylates, α-hydroxycarboxylates, or a mixture of types integrating the characteristics of at least two of them (59). The type of siderophore produced by ZY1 was the same as those produced by *Streptomyces* sp. S073 (23), which is α-hydroxycarboxylate. In addition, ZY1 produces another hydroxamate siderophore. YDE17 secretes only one kind of siderophore, vibrioferrin, according to the previous reports and our present study (41).

*Vibrio* spp., including *V. alginolyticus*, *V. anguillarum*, *V. harveyi,* and *V. parahaemolyticus*, are the main bacterial species that cause vibriosis in shrimp (60). The LD_50_ of YDE17 was similar to that of *V. parahaemolyticus-pir*-201806 (61). To deal with *V. parahaemolyticus* infection, chemicals, antibiotics (62), phages (63), and probiotics (64) have been used. However, antibiotics have harmful effects on the health of consumers because they accumulate in breeding ponds after prolonged use, leading to the development of antibiotic-resistant bacteria (65). Although phages are natural agents, they are not often used, partially because their lytic activity is sensitive to temperature and salinity of culture water (66). As a probiotic, ZY1 offers advantages over antibiotics and phages with high safety, stability to heat, proteinase K, and pH. Compared with the most studied and commonly used probiotic candidates in shrimp aquaculture, such as LAB (67) and *Bacillus* spp. (68), which exhibit an RPS ranging from 51% to 76% (69–71), ZY1 has a relatively high RPS of 83.3%. Its *in vivo* protective effect could also be attributed to the iron deprivation effect, deduced from the higher survival rate of the ZY1/YDE17 group, compared to that of the YDE17/ZY1/FeCl_3_ group. The unexpected lower survival rate of the YDE17/DP group might be due to the limitation in bioavailable iron for both pathogen and hosts (72), given that the shrimps with DP only showed a survival rate of 38%. Thus, *Glutamicibacter* sp. ZY1 may be a preferential candidate for the prevention of *Vibrio* infection.

In conclusion, ZY1, a bacterial isolate from a shrimp farm, exhibited significant inhibitory effects against a diverse of *Vibrio* spp. The inhibitory substances showed stability after thermal, proteinase K, and pH (6–10) treatments. Furthermore, the inhibitory effect of ZY1 and the effect of iron on the inhibitory assay indicated that ZY1 inhibited YDE17 through siderophore production. ZY1 produced the hydroxamate and α-hydroxycarboxylate siderophores, while YDE17 produced vibrioferrin siderophore. All our present results revealed that ZY1 won YDE17 for its higher ability to acquire iron through siderophores, thus exerting inhibitory effects on the growth of YDE17. Finally, artificial infection using shrimp as the experimental animals indicated that ZY1 had the potential to protect shrimp from *V. parahaemolyticus* infection.

## Data Availability

The *Glutamicibacter* sp. ZY1 isolate was deposited into the China General Microbiological Culture Collection (CGMCC, Beijing, China) with accession number CGMCC no. 30798. Additional data will be made available on request.

## SUPPLEMENTAL MATERIAL

Supplemental figures aem.00009-25-s0001.docx Figures S1 to S3.

Table S1 aem.00009-25-s0002.docx Bacterial purification and identification.

ASM does not own the copyrights to Supplemental Material that may be linked to, or accessed through, an article. The authors have granted ASM a non-exclusive, world-wide license to publish the Supplemental Material files. Please contact the corresponding author directly for reuse.
