# Supplementary material for: Glutamicibacter sp. ZY1 antagonizes pathogenic Vibrio parahaemolyticus via iron competition
Source: Appl Environ Microbiol. 2025 Apr 24;91(5):e00009-25. doi: 10.1128/aem.00009-25 (PMC12093950; doi:10.1128/aem.00009-25)

***Glutamicibacter* sp*.* ZY1 antagonizes pathogenic *Vibrio* *parahaemolyticus* via iron competition**

Zhili Shi*^a,b^*, Ya Li*^a,b^*, Weibo Shi*^a,b^*, Zhixin Mu*^a,b^*, Qingxi Han*^a,b,^*, Weiwei Zhang*^a,b,*^*

*^a^* School of Marine Sciences, Ningbo University, Ningbo 315832, P. R. China

^b^ Key Laboratory of Aquacultural Biotechnology Ministry of Education, Ningbo University, Ningbo 315832, P. R. China

^*^Corresponding author: Weiwei Zhang

School of Marine Sciences,

Ningbo University

169 Qixingnan Road, Beilun District, Ningbo, 315832, P. R. China

Tel: +86 15968037766

E-mail: [zhangweiwei1@nbu.edu.cn](mailto:zhangweiwei1@nbu.edu.cn)

**Figure S1**: The inhibitory spectrum of ZY1. A: Bacteria antagonized by ZY1. B: Bacteria could not be antagonized by ZY1. See Table 1 for the taxonomic names of bacteria.


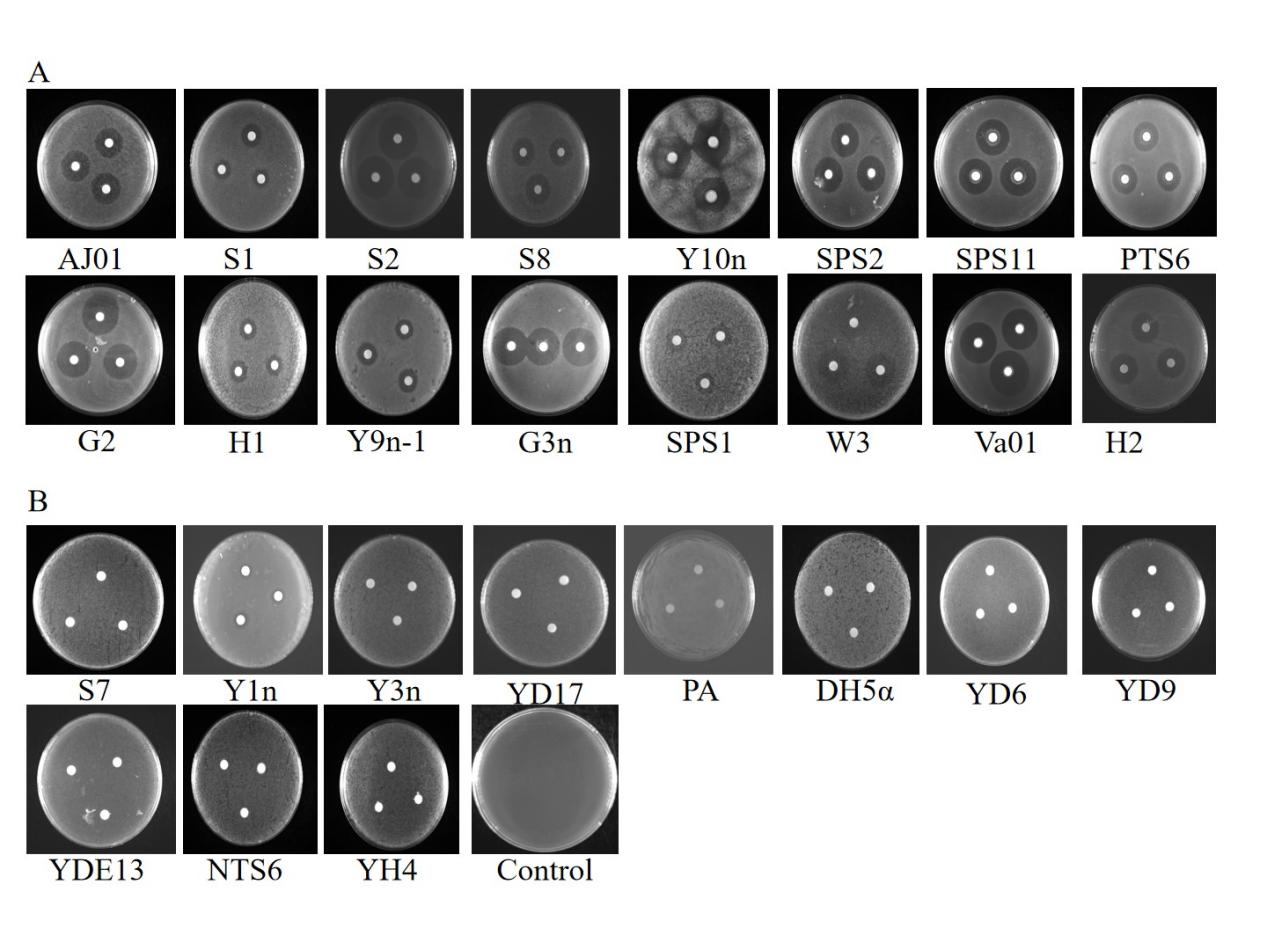


**Figure S2:** ZY1 inhibited YDE17 at different cell ratios. Ten-fold dilution of YDE17 cell suspension were made, and 100 μL of each dilution, 1 × 10^5^ CFU/mL (A), 1 × 10^6^ CFU/mL (B), 1 × 10^7^ CFU/mL (C) and 1 × 10^8^ CFU/mL (D), were spread on 2216E agar plates, respectively. The number of 1, 2, 3, 4, 5, 6, 7, 8 on each plate represented the cell density of ZY1 as 10^1^ CFU/mL, 10^2^ CFU/mL, 10^3^ CFU/mL, 10^4^ CFU/mL, 10^5^ CFU/mL, 10^6^ CFU/mL, 10^7^ CFU/mL, 10^8^ CFU/mL, 10 μL of each dilution was dropped on the filter disc. The dimeter of the inhibitory circle represented ZY1 inhibited YDE17 to varied degrees. Replicates were performed for more than three times from three independent cultures and measurements.


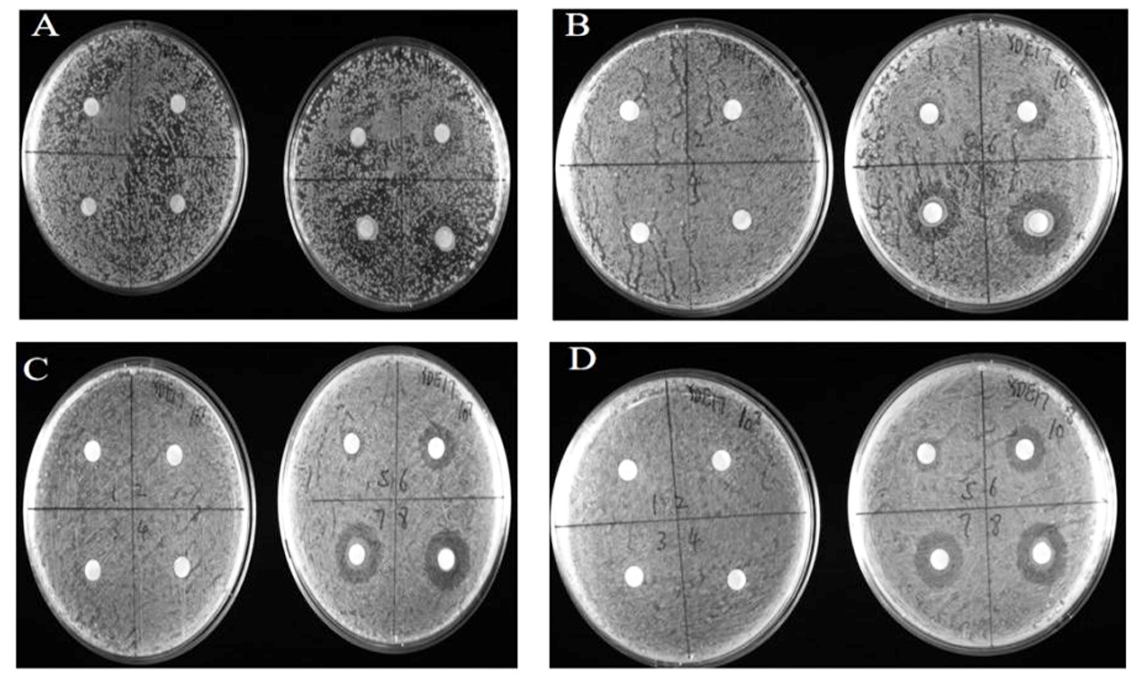


**Figure S3:** Effects of iron on inhibitory activity. A: Effects of different concentrations of FeCl_3_ on the growth of YDE17; B: Inhibitory activity of the cell-free supernatant of ZY1 on YDE17 without FeCl_3_; C: Inhibitory activity of the cell-free supernatant of ZY1 on YDE17 with 200 μM of FeCl_3_; D: Inhibitory activity of the cell-free supernatant of ZY1 on YDE17 with 400 μM of FeCl_3_; E: Inhibitory activity of the cell-free supernatant of ZY1 on YDE17 with 600 μM of FeCl_3_; F: Inhibitory activity of the cell-free supernatant of ZY1 on YDE17 with 800 μM of FeCl_3._ On each plate, there were triplicate filter discs for the same inhibition assay.


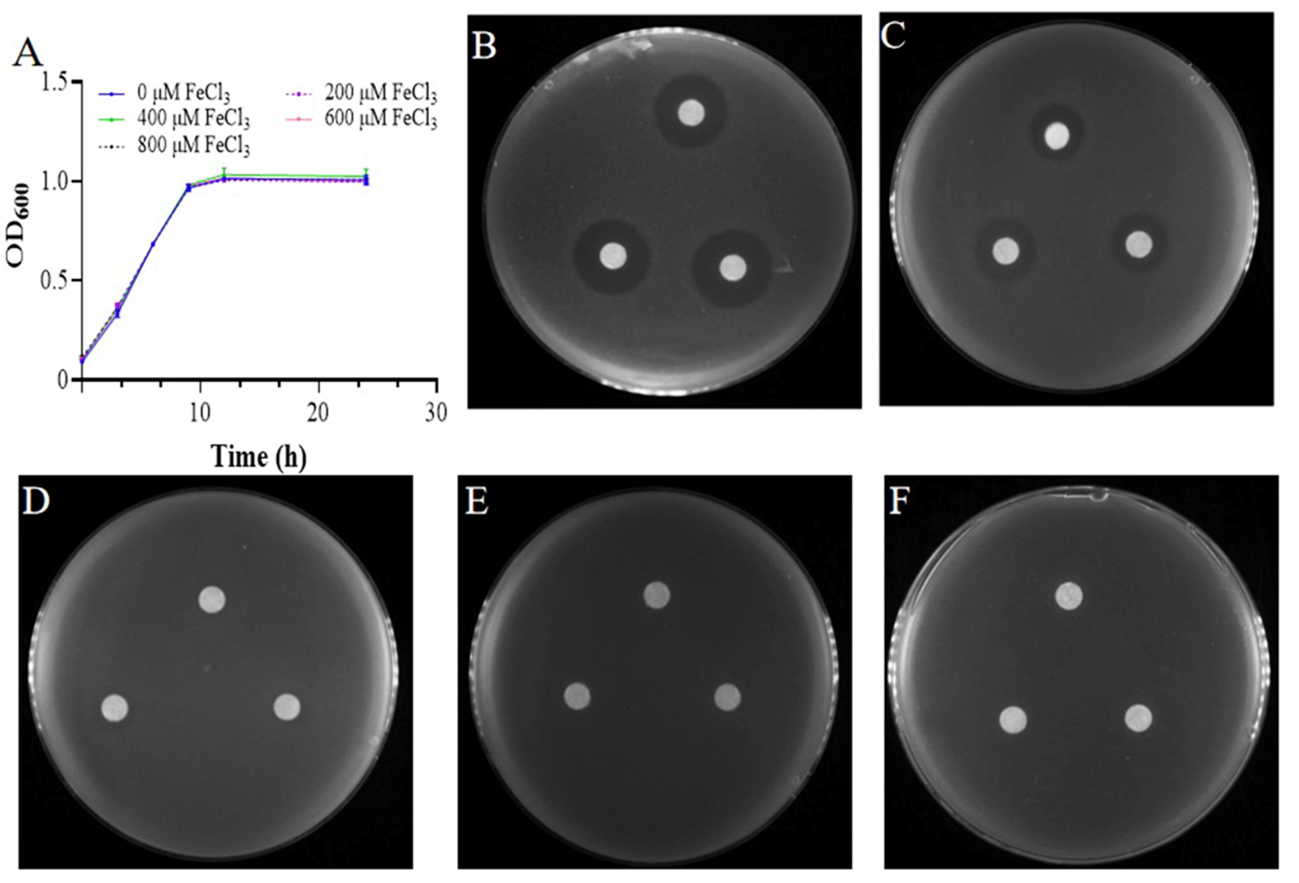

Supplement: Supplemental figures — Figures S1 to S3. [file aem.00009-25-s0001.docx]
