# Supplementary material for: Glutamicibacter sp. ZY1 antagonizes pathogenic Vibrio parahaemolyticus via iron competition
Source: Appl Environ Microbiol. 2025 Apr 24;91(5):e00009-25. doi: 10.1128/aem.00009-25 (PMC12093950; doi:10.1128/aem.00009-25)
Supplement: Table S1 — Bacterial purification and identification. [file aem.00009-25-s0002.docx]

**Table S1**: Bacteria purification and identification.

| **Strain** | **Genus/Species name**  **(with high similarity)** |
| --- | --- |
| Y1, Y8, Y9, Y9-1, Y10, Y11 | *Pseudoalteromonas* *galatheae*, *P*. *piscicida*, *P*. *flavipulchra* |
| Y2, Y7-1 | *P*. *piscicida*, *P*. *flavipulchra*, *P*. *maricaloris* |
| Y3, Y3-1, Y4, Y5 | *P*. *galatheae*, *P*. *piscicida* |
| Y6, Y7-4, Y10-1, Y12, W2-1 | *P*. *piscicida* |
| Y8-1, Y5-1 | *P*. *flavipulchra*, *P*. *maricaloris* |
| Y4-1 | *Pseudoalteromonas* sp, *P*. *piscicida* |
| Y6-1 | *P*. *flavipulchra*, *P*. *piscicida* |
| Y1n, Y2n | *Vibrio* *nereis*, *V*. *hepatarius*, *V*. *corallilyticus* |
| Y3n | *V*. *nereis* |
| Y3n-1, Y4n, Y4n-1, Y8n-1, Y9n-2, Y12n, G2n, S2, W2n-2, W2n-1, W3n | *V*. *chemaguriensis*, *V*. *alginolyticus* |
| Y5n, Y6n, Y7n-1, Y7n-2, Y7n-3, G1, G3, W1, W1n, W2n | *V*. *alginolyticus* |
| Y10n | *V*. *pelagius*, *V*. *fortis*, *V*. *xiamenensis* |
| Y9n-1 | *V*. *natriegens*, *V*. *alginolyticus* |
| G2 | *V*. *alginolyticus*, *V*. *owensii*, *V*. *hyugaensis* |
| G1n | *V.* *chemaguriensis*, *V.* *alginolyticus*, *V.* *natriegen* |
| G3n | *V.* *rotiferianus* |
| S1 | *V.* *furnissii*, *V.* *tritonius*, *V.* *hangzhouensi* |
| W2 | *P.* *galatheae*, *P.* *piscicida*, *P.* *peptidolytica* |
| W3 | *V.* *jasicida*, *V.* *owensii*, *V.* *rotiferianus* |
| ZY1 | *Glutamicibacter* *soli* |
| YDE17 | *V*. *parahaemolyticus* |
